# Metabolomics Analysis in Serum from Patients with Colorectal Polyp and Colorectal Cancer by ^1^H-NMR Spectrometry

**DOI:** 10.1155/2019/3491852

**Published:** 2019-04-07

**Authors:** Jinping Gu, Yaqing Xiao, Dan Shu, Xianrui Liang, Xiaomin Hu, Yuanyuan Xie, Donghai Lin, Hua Li

**Affiliations:** ^1^Key Laboratory for Green Pharmaceutical Technologies and Related Equipment of Ministry of Education, College of Pharmaceutical Sciences, Zhejiang University of Technology, Hangzhou, China; ^2^The First Affiliated Hospital of Xiamen University, Xiamen University, Xiamen, China; ^3^Department of Pathology, University of Hong Kong, Hong Kong; ^4^Collaborative Innovation Center of Yangtze River Delta Region Green Pharmaceuticals, Zhejiang University of Technology, Hangzhou, China; ^5^High-Field NMR Research Center, MOE Key Laboratory of Spectrochemical Analysis & Instrumentation, College of Chemistry and Chemical Engineering, Xiamen University, Xiamen, China

## Abstract

Colorectal cancer (CRC) is one of the leading causes of cancer-related death worldwide. Colorectal adenomatous polyps are at high risk for the development of CRC. In this report, we described the metabolic changes in the sera from patients with colorectal polyps and CRC by using the NMR-based metabolomics. 110 serum samples were collected from patients and healthy controls, including 40 CRC patients, 32 colorectal polyp patients, and 38 healthy controls. The metabolic profiles and differential metabolites of sera were analyzed by multivariate statistical analysis (MSA), including principal component analysis (PCA), partial least squares discriminant analysis (PLS-DA), and orthogonal partial least squares discriminant analysis (OPLS-DA) methods. A total of 23 differential metabolites were identified from MSA. According to the pathway analysis and multivariate ROC curve-based exploratory analysis by using the relative concentrations of differential metabolites, we found abnormal metabolic pathways and potential biomarkers involved with the colorectal polyp and CRC. The results showed that the pyruvate metabolism and glycerolipid metabolism were activated in colorectal polyps. And the glycolysis and glycine, serine, and threonine metabolism were activated in CRC. The changed metabolism may promote cellular proliferation. In addition, we found that the rates of acetate/glycerol and lactate/citrate could be the potential biomarkers in colorectal polyp and CRC, respectively. The application of ^1^H-NMR metabolomics analysis in serum has interesting potential as a new detection and diagnostic tool for early diagnosis of CRC.

## 1. Introduction

Colorectal cancer (CRC) is one of the most prevalent digestive system malignant tumors worldwide. The occurrence of tumors is multistep and multifactorial, including gene mutation, genetic, immune. According to the relevant date, the morbidity and mortality of CRC are second only to lung cancer and breast cancer [1]. As the third most common type of cancer in the US according to the American Cancer Society, over 136000 new CRC cases and 50000 deaths are estimated for the year 2015 [2]. In China, with the improvement of living standards and changes in diet, CRC mortality rapidly increased to become the fifth most common cause of cancer-related deaths in 2012 and continued to rise [1].

At present, CRC early assessment and diagnostic methods mainly include digital rectal exam, fecal occult blood test (FOBT), serum tumor marker detection, sigmoidoscopy, colonoscopy, virtual colonoscopy, and double-contrast barium enema (DCBE). Each has its own advantages and disadvantages. Patients with colon polyps are at high risk for the development of colon cancer. However, the only colonoscopy has sufficient sensitivity to detect polyps. While colonoscopy and sigmoidoscopy remain the most accurate methods for screening and diagnosis of CRC and polyps, they have significant disadvantages, including invasiveness, potential hazards of postoperative complications, and high fee [3, 4]. Of the CRC patients, only 40% are diagnosed and treated in the early stage [5]. Therefore, new, highly sensitive, specific, noninvasive, and robust screening methods are urgently needed for the early diagnosis of CRC.

Metabolomics is the “systematic study of unique chemical fingerprints that specific cellular processes leave behind” [6]. The metabolome represents the collection of all metabolites in a biological cell, tissue, organ, or organism, which are the final products of cellular processes [7]. Metabolomics could supply indispensable information to provide a better understanding of cellular biology in the system biology and functional genomics [8, 9]. ^1^H nuclear magnetic resonance (^1^H-NMR) is one of the major analytical methods of metabolomics. Recently, NMR-based metabolomics is widely used in cancer diagnosis and prognosis [10–14]. In addition, metabolomics studies of CRC patients have found some potential biomarkers for CRC detection and prognosis [15, 16]. The colorectal adenomatous polyp is a precancerous lesion of CRC; however, a few studies have focused on identifying metabolite changes between CRC and colorectal polyps.

In this study, we have utilized NMR-based metabolomics combined with multivariate statistical analysis (MSA), to investigate differential metabolic profiles between sera from CRC patients, colorectal polyp patients, and healthy controls. In this study, we are looking forward to finding out the differential metabolites associated intimately with CRC, as the potential biomarkers for detecting between the CRC and colorectal polyp patients.

## 2. Materials and Methods

### 2.1. Chemicals

D_2_O (99.9% D) was purchased from Sigma-Aldrich (St. Louis, MO). K_2_HPO_4_ and NaH_2_PO_4_ were purchased from Xilong Chemical Co. Ltd. (Guangdong, China). Phosphate buffer solution (pH 7.4) was prepared by 50 mM K_2_HPO_4_/NaH_2_PO_4_ in the D_2_O.

### 2.2. Serum Sample Collection

CRC and colorectal polyp patients were recruited from the Department of Gastroenterology and Oncological Surgery of the First Affiliated Hospital of Xiamen University. Healthy controls were recruited from the physical examination center of the First Affiliated Hospital of Xiamen University. All of the CRC patients and colorectal polyp patients had been confirmed by colonoscopy and histology. The participating subjects were recruited for this study, as summarized in Table S1. Blood samples (5 mL) were collected from CRC patients, colorectal polyp patients, and healthy controls, who were on a rice gruel for at least 48 hours. Blood was allowed to clot at room temperature for 1 hour before centrifugation (4°C, 4500 g, 15 min). Then, the serum (supernatant) was carefully separated, collected in cryovials, and stored in a -80°C refrigerator.

### 2.3. Preparation of Samples and Acquisition for ^1^H-NMR Spectroscopy

Before NMR analysis, we followed the methods of Gu et al. [17] to prepare the sera. The sera were thawed in ice, and 300 *μ*L aliquots were mixed with 210 *μ*L PBS to minimize variations in pH. Then, all samples were centrifuged at 12000 g for 10 min at 4°C and transferred into a 5 mm NMR tube. All ^1^H-NMR spectra were acquired at 298 K on the Bruker AVANCE III at 600 MHz. One-dimensional spectra were acquired by using the Carr-Purcell-Meiboom-Gill (CPMG) pulse sequence (RD − 90^o^ − (*τ* − 180^o^ − *τ*)_*n*_ − ACQ) with water suppression. We set up the total spin-spin relaxation delay as 80 ms to attenuate broad NMR signals of macromolecules and retain signals of metabolites, according to differences of *T*_2_ relaxation times from macromolecules and metabolites. The spectral width was 20 ppm with an acquisition time of 1.64 s, and a total of 256 free induction decays were collected into 64 k data points for each spectrum.

### 2.4. Spectral Processing and Multivariate Statistical Analysis (MSA)

Before Fourier transformation, the free induction decay (fid) data was multiplied by an exponential line-broadening function of 0.3 Hz. The NMR spectra were manually phased, corrected for baseline correction, referenced to the lactate (CH_3_, at *δ*1.33 ppm), and carefully aligned using MestReNova (version 6.1, MestReLab Research S.L., Spain). The spectral region of *δ*0.0-9.0 ppm was segmented into 9000 bins with a width of 0.001 ppm. The residual integrals from the region of *δ*4.6-5.1 ppm in suppressed water resonance were excluded in all spectra. Each sample data was normalized to the sum of the spectral intensity to compensate for differences in the concentrations of samples [18].

Subsequently, the normalized data were subjected into MSA by using the SIMCA-P^+^ 13 software package (Umetrics, Umeå, Sweden). The principal component analysis (PCA) model approximates the variation in a data table by a low-dimensional model plane. Then, the partial least squares discriminant analysis (PLS-DA) [19] and orthogonal signal correction partial least squares discriminant analysis (OPLS-DA) [20] were used to classify the samples and extract the correlated variables in relevance with the sample belongings. Both PLS-DA and OPLS-DA were also operated by using the SIMCA-P^+^ 13 software package. As the supervised MSA (PLS-DA and OPLS-DA), the response permutation testing (RPT) was carried out to measure the robustness of the model [21]. Furthermore, the CV-ANOVA was also used to measure the robustness of the OPLS-DA models [22].

### 2.5. Identification of Differential Metabolites

In the OPLS-DA model, most of the variables related to the class belongings were described in the first principal component prediction [20]. Two critical parameters were used to identify the differential metabolites. One is the variable importance in the projection (VIP) from the OPLS-DA model, which sorts the importance of each variable for classification of the metabolic profiles. When VIP > 1, the variables were considered statistically significant variables [23, 24]. The other is the correlation coefficients of the variables relative (*r*) in the OPLS-DA models. According to degrees of freedom (*X* − 1), where *X* is the smaller number of n1 and n2 in OPLS-DA models [25], the threshold values were calculated for identification of the differential metabolites. The reconstitution loading plots of the OPLS-DA model were created in MATLAB (version 2011b, MathWorks Inc., USA).

### 2.6. Compared with the Most Relevant Pathways by Using the Pathway Analysis

For a better and more complete understanding of the metabolic changes, the metabolic pathway analysis was applied to find the most relevant pathways involved with the CRC and colorectal polyp. Before the pathway analysis, we calculated the relative concentration of the differential metabolites. The pathway analysis was carried out on the web server of MetaboAnalyst 3.0 [26]. In the pathway analysis module of MetaboAnalyst 3.0, there are two parameters to determine the relevant pathways involved with CRC and colorectal polyp. One parameter is the statistical *p* values from the quantitative enrichment analysis [27], and the other is the pathway impact value, which is calculated by the topological analysis with the relative betweenesss centrality..

### 2.7. Potential Biomarker Discovery by Using the Multivariate Receiver Operating Characteristic (ROC) Analysis

Metabolomics has proved to be useful in the biomarker discovery of cancer in early diagnostic [28, 29]. In our study, we used the multivariate ROC analysis in MetaboAnalyst 3.0 [26] to find the potential biomarkers from differential metabolites. ROC curves compare sensitivity versus specificity across a range of values for the ability to predict a dichotomous outcome. In the ROC curve, sensitivity refers to the percentage of subjects with target conditions and positive results; meanwhile, specificity is the percentage of subjects without target conditions and negative results [30]. In the biomarker analysis module of the web server of MetaboAnalyst 3.0, the feature ranking method with Random forest algorithm [31] is applied to select the potential biomarkers.

## 3. Results

### 3.1. Metabolic Profile Analysis of Colorectal Polyp and CRC Patients

The typical ^1^H-NMR spectra of sera from the three groups were showed in Figure S1. A number of metabolites were assigned based on previous literatures [32, 33] and confirmed by public NMR database (Human Metabolome Database, version 3.0, http://www.hmdb.ca/) [34]. Further, these metabolites were confirmed with a 2D ^1^H-^1^H TOCSY spectrum of a control serum (Figure S2).

**Figure 1:**
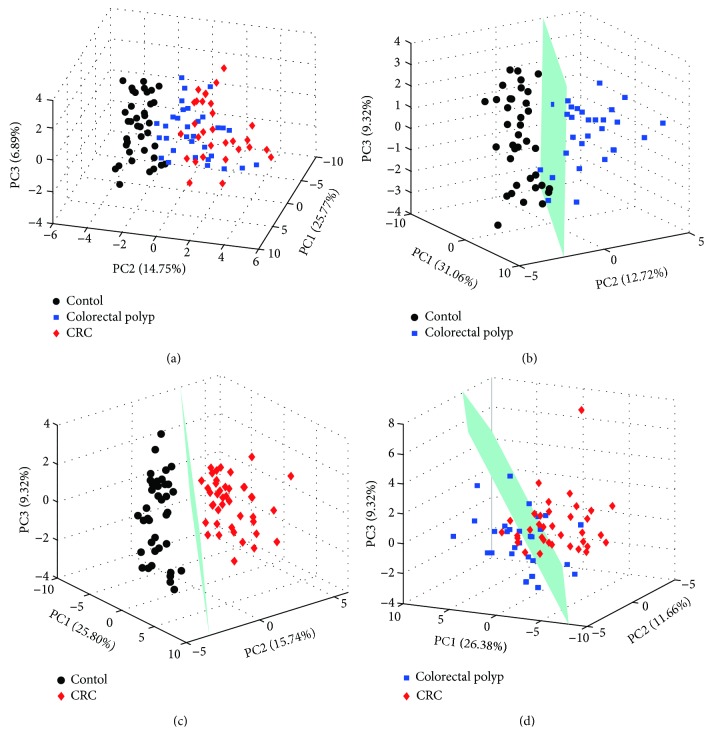
PCA score plots derived from NMR spectra of the serum samples. (a) All samples; (b) colorectal polyps vs. controls; (c) CRC vs. control; (d) CRC vs. colorectal polyps.

For comprehensive observation of the metabolic profiles from the three groups, the PCA was performed on the respective NMR data of sera. The analysis results of PCA were shown in Figure 1. The metabolic profiles of colorectal polyp and CRC could be distinguished from those of the healthy control in the PCA score plot (Figure 1(a)) with the first three principal components (PC1, PC2, and PC3). In the PCA models, the metabolic profiles of colorectal polyp could be basically differentiated from those of the control (Figure 1(b)). CRC is metabolically differentiated from the control obviously (Figure 1(c)). However, the groups of colorectal polyp and CRC displayed separations with partial overlap in the score plot (Figure 1(d)).

To assess the variations between the groups, PLS-DA with the first two predicted principal components (tp1 and tp2) would like to be utilized. In the score plots of PLS-DA models (Figure S3 A, B, and C), the metabolic profiles of each could be distinguished between each other. The validation plots of these corresponding RPTs (Figure S3 D, E, and F) indicated that the classifications were reliable. Furthermore, the corresponding RPTs and CV-ANOVAs of OPLS-DA models were also used to measure the robustness of these OPLS-DA models (Figure S4, Table S2-S4).

**Figure 2:**
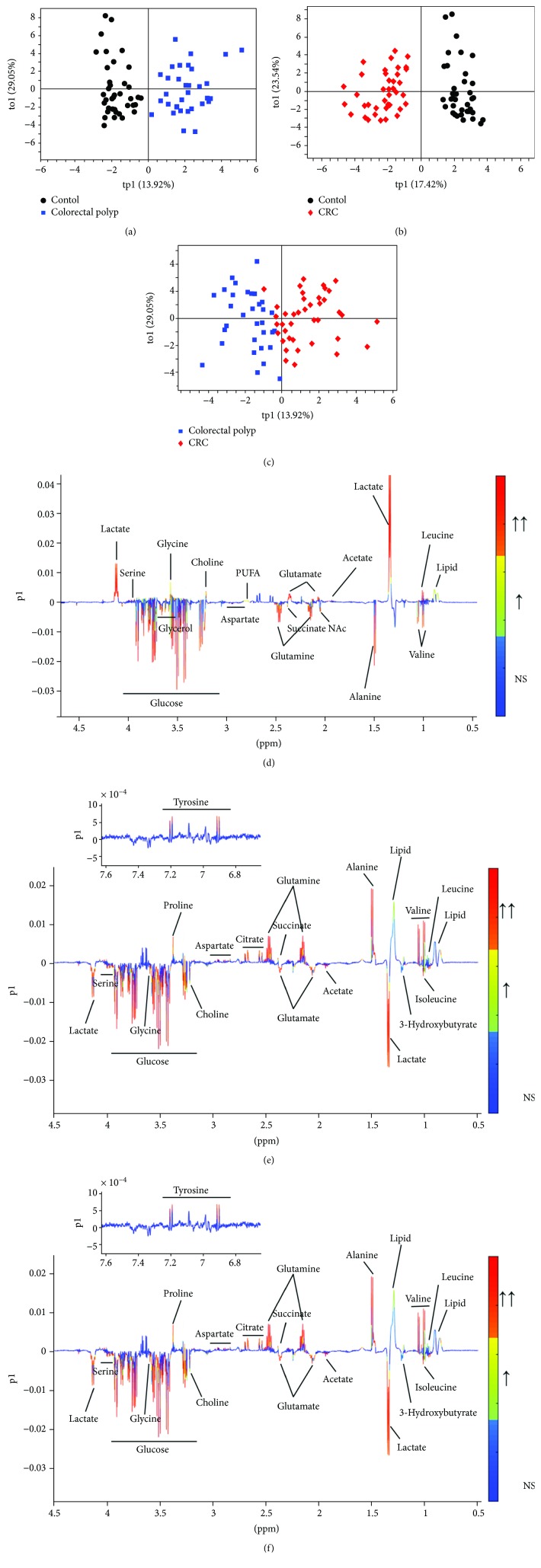
(a–c) OPLS-DA score plots derived from NMR spectra of serum samples and (d–f) the corresponding loading plots used to identify differential metabolites. (a, d) Colorectal polyps vs. controls; (b, e) CRC vs. control; (c, f) CRC vs. colorectal polyps. The gradient red color indicates that the variables are very significant (∣*r*∣ > 0.442 in (d), ∣*r*∣ > 0.408 in (e), and ∣*r*∣ > 0.442 in (f); VIP > 1); gradient orange indicates that the variables are significant (0.344 < ∣*r*∣ < 0.442 in (d), 0.316 < ∣*r*∣ < 0.408 in (e), and 0.344 < ∣*r*∣ < 0.442 in (f); VIP > 1); blue indicates that the variables are insignificant (NS).

The differential metabolites which are significant responsible for distinguishing these three groups were identified in the OPLS-DA loading plots. In the OPLS-DA models, the score plots showed separations between each other (Figures 2(a)–2(c)). The corresponding loading plots offered an insight into the types of metabolites on the first principal component according to the VIPs and correlation coefficients (Figures 2(d)–2(f)). According to the OPLS-DA model of the colorectal polyp group compared to that of the control group, the levels of lipid, leucine, lactate, acetate, glutamate, PUFA, choline, glycine, and betaine were increased in the colorectal polyp group, and the levels of valine, alanine, N-acetyl glycoproteins, glutamine, succinate, aspartate, glycerol, and glucose were decreased in the colorectal polyp group (Figures 2(a) and 2(d)). In the model of the CRC group compared to that of the control group, the levels of isoleucine, 3-hydroxybutyrate, lactate, acetate, glutamate, choline, glycine, serine, and glucose were increased in the CRC group, and the levels of lipid, leucine, valine, alanine, glutamine, succinate, citrate, aspartate, proline, and tyrosine were decreased in the CRC group (Figures 2(b) and 2(e)). There are some different metabolites between the colorectal polyp group and CRC group. The metabolites of lysine, N-acetyl glycoproteins, glutamine, glycerol, serine, and glucose were elevated in the CRC group. The metabolites of lipid, leucine, valine, alanine, glutamate citrate, PUFA, proline, and tyrosine were reduced in the CRC group (Figures 2(c) and 2(f)). The detailed information of these metabolites was listed in Table 1.

### 3.2. Major Pathways with Concerted Alterations in the Colorectal Polyp and CRC

**Figure 3:**
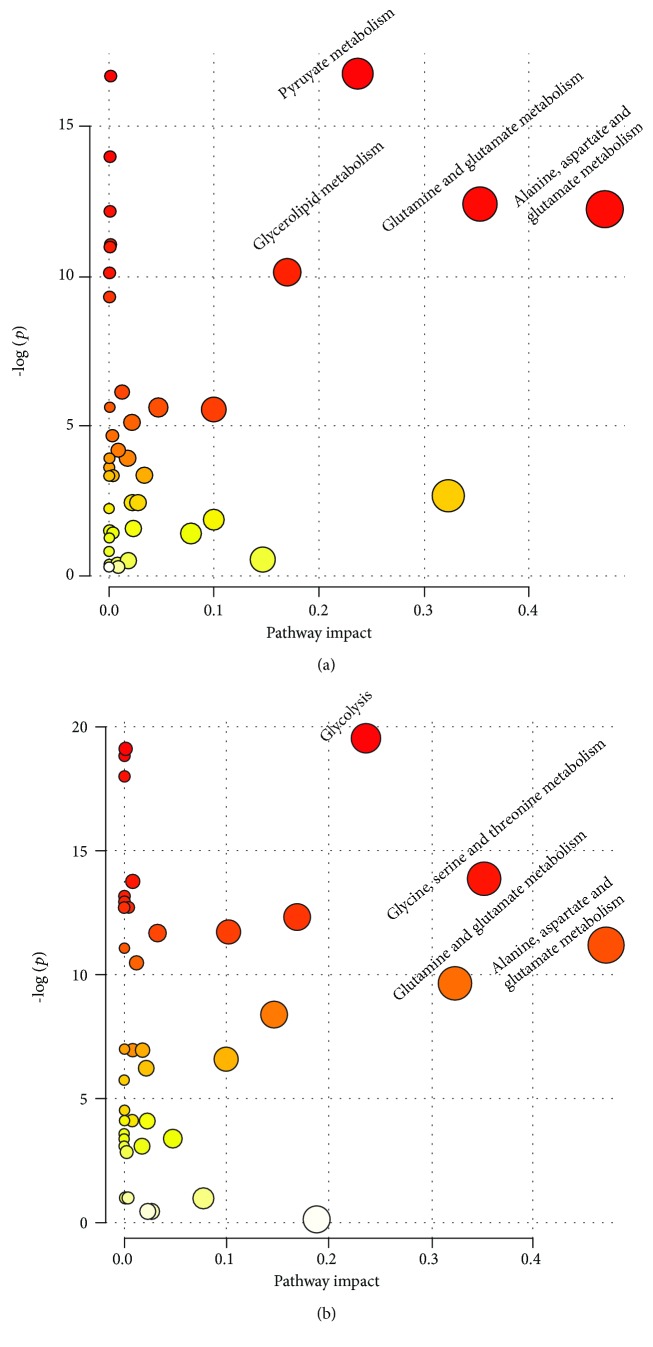
The aberrant metabolic pathways using the relative concentrations of differential metabolites from NMR spectra of serum samples in the pathway analysis module of MetaboAnalyst 3.0. (a) Colorectal polyps vs. controls; (b) CRC vs. control.

In the colorectal polyp, the major relevant pathways were the pyruvate metabolism, glycerolipid metabolism, glutamine and glutamate metabolism, and alanine, aspartate, and glutamate metabolism (Figure 3(a)). Then, the major relevant pathways changed in the CRC. The major pathways were glycolysis; glycine, serine, and threonine metabolism; glutamine and glutamate metabolism; and alanine, aspartate, and glutamate metabolism (Figure 3(b)).

### 3.3. Potential Biomarkers in the Colorectal Polyp and CRC

**Figure 4:**
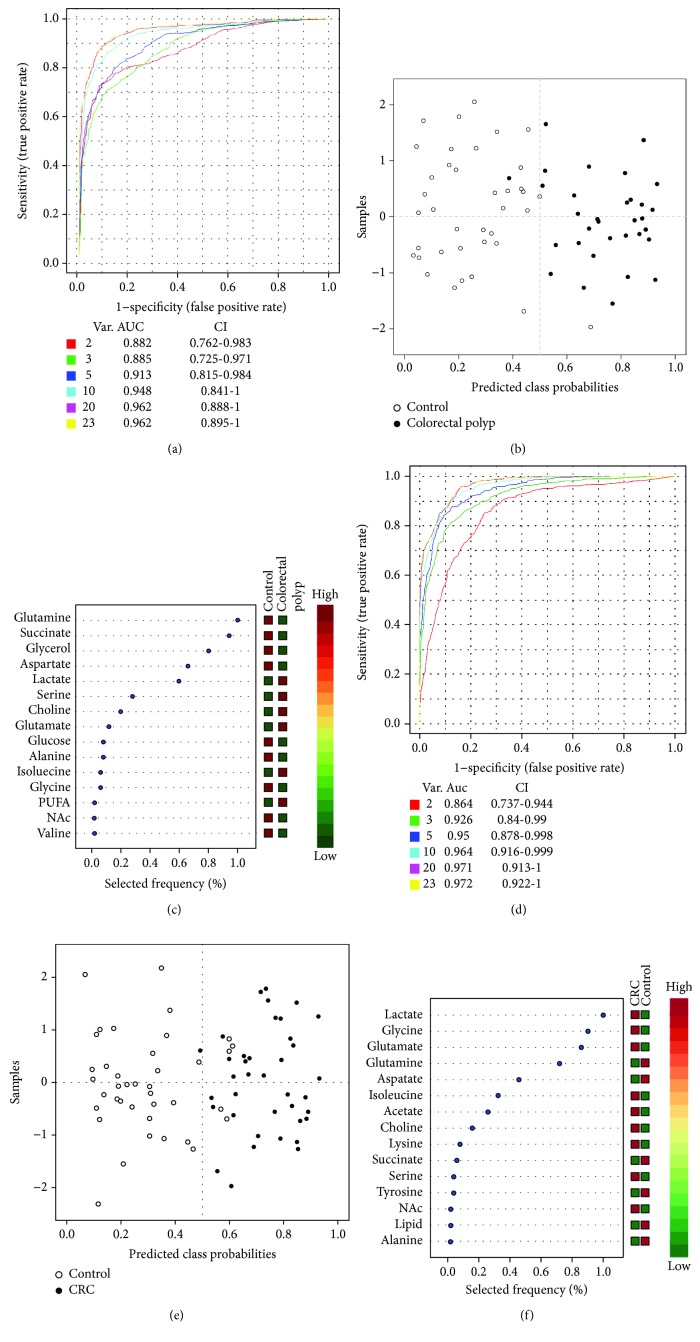
The results of the important feature identification in the serum data. (a) The multivariate ROC curves based on the cross-validation to determine the features (5 features); (b) the predicted class probability plots (average of the cross-validation) for each sample using the base classifier (based on AUC with 5 features). (c) Rank features by the selected frequency of being selected.

Using the biomarker analysis from the web server of MetaboAnalyst 3.0, we found some potential biomarkers in the colorectal polyp and CRC. At first, we performed multivariate ROC curve analyses based on the Random forest algorithms. The results of multi-ROC curve analyses showed that the models with five features both in colorectal polyp and CRC data have a good discriminant ability (Figures 4(a) and 4(d)). The predicted class probabilities for each sample using the classifier of five feature models (Figures 4(b) and 4(e)) also verified that these five features could distinguish the colorectal polyp and CRC samples from the control samples. The results of the feature ranking showed the potential biomarker ranking (Figures 4(c) and 4(f)). In the colorectal polyp data, the different metabolites of glutamine, succinate, glycerol, aspartate, and lactate were the potential biomarkers (Figure 4(c)). In the CRC data, the different metabolites of lactate, glycine, glutamate, glutamine, and aspartate were the potential biomarkers (Figure 4(f)). These potential biomarkers could be transformed into the early diagnostic index of colorectal polyp and CRC.

## 4. Discussion

Metabolic polymorphisms in human carcinogenesis derived from the altered oncogenic expression, variable hypoxia levels, and the utilization of different carbon sources may produce diverse metabolic phenotypes and treatment responses [35]. Toward the goal of a system view of the metabolic changes in CRC, we have therefore researched metabolic changes in the sera from the colorectal polyp and CRC patients and healthy control volunteers.

In this study, we found that the metabolic profiles of these three groups could be distinguished by using the NMR-based metabolomics combined with multivariate statistical analysis. The similar results were also observed by others. Zhu et al. found that metabolites are obviously different between the serum samples of these three groups by using LC-MS [36]. In addition, Ong et al. also found this metabolic pathway was significant change between the tissure of CRC and adjacent matched normal mucosa by using the GC-MS and LC-MS/MS [37].

### 4.1. Metabolic Changes in the Colorectal Polyp

Comparing to the healthy control, we focused on the altered metabolism in the colorectal polyp. The major abnormal metabolic pathways were the pyruvate metabolism, glycerolipid metabolism, glutamine and glutamate metabolism, and alanine, aspartate, and glutamate metabolism. Pyruvate metabolism predominates reliance on carbohydrate metabolism for ATP generation [38]. It is also involved in carbon flux to regulate the ATP generation [39]. Lactate and acetate are the key metabolites in the pyruvate metabolism. In our data of colorectal polyp, the level of lactate was increased and the level of acetate was decreased. The changes of these metabolites also suggested that the pyruvate metabolism was abnormal in the colorectal polyp. The glycerolipid metabolism was the anther abnormal metabolic pathway. The increased levels of lipid and PUFA and the decreased level of glycerol could also prove that the glycerolipid metabolism was abnormal in the colorectal polyp. This phenomenon suggested that the glycerolipid metabolism participates in the ATP generation. Two other abnormal metabolic pathways were focused on the amino acid metabolism. The increased level of glutamate and the decreased level of glutamine implied that the relevant oxidative stress was activated in the colorectal polyp. Crespo-Sanjuán et al. also verified that the oxidative stress level was increased in the serum of polyp patients [40]. Alanine, aspartate, and glutamate metabolism was abnormal in polyps. The levels of alanine and aspartate were decreased in the serum of polyps. The alanine is the product of pyruvate metabolism. Aspartate is one of the important amino acids for the biosynthesis of the building block [41]. Chen et al. found the metabolic profile differences between colorectal polyp patients and controls [4]. Using the seemingly unrelated regression in the NMR data of sera, they found that the alanine, aspartate, and glutamate metabolism was abnormal in the polyps.

### 4.2. Metabolic Changes in CRC

Comparing to the controls, the major abnormal metabolic pathways were the glycolysis; glycine, serine, and threonine metabolism; glutamine and glutamate metabolism; and alanine, aspartate, and glutamate metabolism. Glycolysis is the important part of carbon flux in cell proliferation [42]. In our work, the level of lactate, which is the terminal product of glycolysis, was increased in the sera of CRC and citrate and succinate (intermediate products of the citrate cycle) were decreased. These changed metabolisms were known as the “Warburg effect” [43]. The “Warburg effect” is known to be a characteristic feature of cancer metabolism, which describes the increased rate of glycolysis during tumor growth. Previous studies have also found that compared with the control group, the level of lactate was significantly increased in the serum and tissue samples from CRC patients and the intermediates of citrate cycle levels were decreased [44–46]. The levels of glycine and serine were increased in the sera of CRC, and the level of serine in CRC was even higher than that in the colorectal polyp. Serine is one of the important amino acids in cancer metabolism [47]. Serine could be involved into the glycolysis by deriving from 3-phospho-D-glycerate, which is an intermediate of glycolysis [48]. Serine could transform into glycine, which is associated with cancer cell proliferation [49]. The increased levels of glycine and serine may imply that the activated glycine, serine, and threonine levels could be a feature of the metabolic pathway in the CRC. The two other abnormal metabolic pathways in CRC were the same as those in the colorectal polyp. The change of metabolites (glutamine, glutamate, alanine, and aspartate) involved with these two pathways was similar to that between colorectal polyp and CRC patients.

### 4.3. Metabolism Similarities and Differences between Colorectal Polyp and CRC

Compared to the abnormal metabolic pathways and changed metabolites between colorectal polyp and CRC, we found some similarities and differences in metabolism. Outside of the two abnormal metabolic pathways (glutamine and glutamate metabolism and alanine, aspartate, and glutamate metabolism), the changed choline was alike between colorectal polyp and CRC. In our work, the level of choline was significantly increased in sera of colorectal polyp and CRC. The elevation of choline-related metabolites in tumors probably resulted from metabolism of the membrane lipids due to accelerated cell proliferation [50, 51].

However, we found that the level of N-acetyl glycoprotein was different in the colorectal polyp and CRC patients. The N-acetyl glycoprotein was increased in the sera of colorectal polyps and not significantly changed in those of the CRC. N-acetyl glycoprotein contains N-acetyl cysteine, while N-acetyl cysteine is a precursor of glutathione (GSH) synthesis [52]. Because the level of N-acetyl glycoprotein was decreased in the serum of colorectal polyps, N-acetyl cysteine was also reduced accordingly, so that the equilibrium of oxidation and antioxidation may be disordered and then cause the body damage. The other differences in metabolites were 3-hydroxybutyrate, lipid, PUFA, glycerol, and glucose. The metabolites of 3-hydroxybutyrate, lipid, PUFA, and glycerol were involved with the glycerolipid metabolism, which was abnormal in the colorectal polyp. Glucose was involved with the glycolysis, which was abnormal in CRC. These phenomena suggested that the ATP generation from carbon flux was different between colorectal polyp and CRC.

In addition, we found that 3-hydroxybutyrate was only significantly increased in the sera of CRC patients. 3-Hydroxybutyrate is an end product of fatty acid *β*-oxidation. Its high level and lipid and glycerol low levels may suggest that the cancer cells enhanced fatty acid *β*-oxidation to support the energy demand of cancer cell proliferation. The activated fatty acid *β*-oxidation has been confirmed in previous proteomics research [53]. This metabolite had the similar trend as that in the research of Qiu et al. [54].

### 4.4. Diagnostic Potentials of Potential Biomarkers from Differential Metabolites

The differential metabolites found in serum samples could be used as candidate biomarkers to investigate their diagnostic potential using the sera as the samples. According to the results from the multi-ROC analysis, we found that glutamine, succinate, glycerol, aspartate, and lactate were the potential biomarkers in colorectal polyp and lactate, glycine, glutamate, glutamine, and aspartate were the potential biomarkers in CRC.

**Figure 5:**
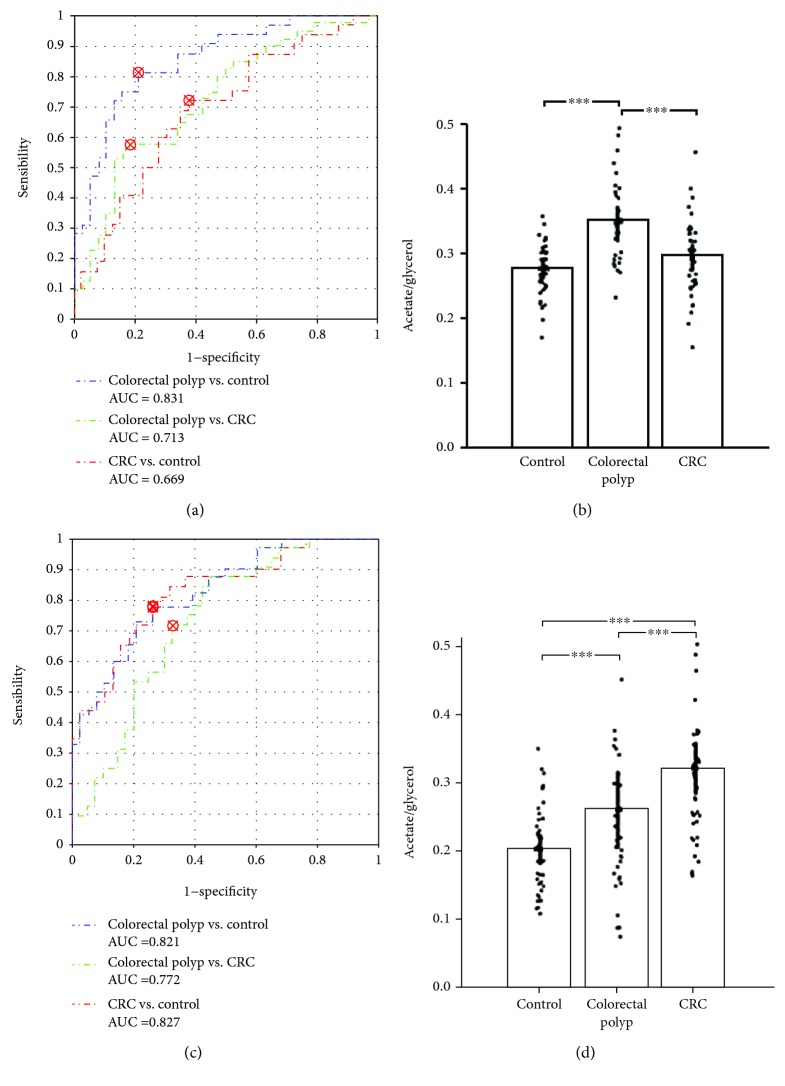
Diagnostic potential for colorectal polyp and CRC by rates (acetate/glycerol, lactate/citrate) from serum data. (a) ROC curves of the rate of acetate/glycerol (colorectal polyp vs. control with a 0.831 AUC, colorectal polyp vs. CRC with a 0.713 AUC, and CRC vs. control with a 0.669 AUC); (b) rate of acetate/glycerol in serum samples from control, colorectal polyp, and CRC patients; (c) ROC curves of the rate of lactate/citrate (colorectal polyp vs. control with a 0.821 AUC, colorectal polyp vs. CRC with a 0.772 AUC, and CRC vs. control with a 0.827 AUC); (d) rate of lactate/citrate in serum samples from control, colorectal polyp, and CRC patients. ^∗∗^ *p* < 0.01; ^∗∗∗^ *p* < 0.001.

Combining with the results of pathway analysis and the multi-ROC analysis, the metabolites involved with glycerolipid metabolism may become the potential biomarkers for colorectal polyps. Then, the metabolites involved with glycolysis may be the potential biomarkers for CRC. In order to better distinguish the colorectal polyps from others, we used the rate between the acetate and glycerol (acetate/glycerol) as the discriminative mark. The result indicated that this rate was efficient for distinguishing colorectal polyps from others (colorectal polyp vs. control with a 0.831 AUC, colorectal polyp vs. CRC with a 0.713 AUC, and CRC vs. control with a 0.669 AUC; Figure 5(a)). The rates of lactate and citrate (lactate/citrate) were deemed as the discriminative mark for distinguishing these three groups (colorectal polyp vs. control with a 0.821 AUC, colorectal polyp vs. CRC with a 0.772 AUC, and CRC vs. control with a 0.827 AUC; Figure 5(c)). The cutoff points are also marked on the ROC curves (Figures 5(a) and 5(c)). The detailed parameters were shown in Tables S5-S6. These parameters proved the discriminant ability of these rates. To go a step further and validate the testing of the diagnostic effect of these rates, we used the support vector machine (SVM) classifier to verify the diagnostic effect by using a new validation set. The detailed information of validation samples was listed in Table S7. The results showed that the rates of acetate/glycerol and lactate/citrate have good discriminant abilities (Figure S5). In addition, we needed more patients to confirm the effectiveness for colorectal polyps and CRC diagnosis in the future.

In conclusion, the metabolic profile analysis of sera provided a holistic view of the metabolic phenotypes of colorectal polyps and CRC patients. According to the based ^1^H-metabolomics analysis, the differential metabolites were identified in the sera. On the basis of the pathway analysis, the abnormal metabolic pathways were confirmed in the sera from colorectal polyp and CRC patients compared to the controls. The pathways of glutamine and glutamate metabolism and alanine, aspartate, and glutamate metabolism were abnormal in the colorectal polyps and CRC. The pyruvate metabolism and glycerolipid metabolism were activated in colorectal polyps. Moreover, the glycolysis and glycine, serine, and threonine metabolism were activated in CRC. The changed metabolism may promote cellular proliferation. The rapid consumption of energy by the upregulated glycolysis is consistent with that of the Warburg effect.

The diagnostic potential marks of the rates of acetate and glycerol in the colorectal polyps and the rates of lactate and citrate in CRC were found in the serum samples on the basis of the results of pathway analysis and multi-ROC analysis. These rates have been validated in the ROC curve in distinguishing the colorectal polyp and CRC patients. In the future, more serum samples are needed for the verification of these rates as biomarkers in clinical diagnosis.

## Figures and Tables

**Table 1:** Changes in relative levels of metabolites in the serum samples from CRC patients, colorectal polyp patients, and healthy controls.

Metabolites	*δ* (^1^H)	Colorectal polyp vs. control			CRC vs. control			CRC vs. colorectal polyp		
		VIP	∣*r*∣	Change/fold change	VIP	∣*r*∣	Change/fold change	VIP	∣*r*∣	Change/fold change
Lipid	0.83-0.89 (bra)	2.51	0.41	↑^∗^/1.07	2.15	0.33	↓^∗^/1.19	2.24	0.36	↓^∗^/1.15
Leucine	0.96 (t), 1.70 (m), 3.73 (m)	1.3	0.52	↑^∗∗^/1.08	1.16	0.45	↓^∗∗^/1.23	2.04	0.45	↓^∗∗^/1.16
Isoleucine	9.92 (t), 1.02 (d), 3.73 (m)	—	—	—/1.01	1.34	0.53	↑^∗∗^/1.28	—	—	—/1.01
Valine	9.78 (d), 1.05 (d), 3.61 (d)	1.92	0.54	↓^∗∗^/1.09	3.77	0.50	↓^∗∗^/1.17	3.03	0.35	↓^∗^/1.12
3-Hydroxybutyrate	1.20 (d), 2.28 (q), 2.40 (q), 4.15 (m)	—	—	—/1.01	1.29	0.47	↑^∗∗^/1.59	—	—	—/1.08
Lactate	1.33 (d), 4.12 (q)	9.52	0.63	↑^∗∗^/1.51	4.6	0.67	↑^∗∗^/1.48	—	—	—/1.06
Alanine	1.48 (d), 3.78 (q)	1.13	0.56	↓^∗∗^/1.12	1.65	0.48	↓^∗∗^/1.17	2.19	0.36	↓^∗^/1.18
Acetate	1.92 (s)	1.46	0.57	↑^∗∗^/1.21	1.25	0.54	↑^∗∗^/1.18	—	—	—/1.02
Glutamate	2.08 (m), 2.34 (m)	3.19	0.86	↑^∗∗^/1.41	1.95	0.72	↑^∗∗^/1.20	1.51	0.38	↑^∗^/1.18
Glutamine	2.13 (m), 2.45 (m)	5.19	0.82	↓^∗∗^/1.19	2.74	0.67	↓^∗∗^/1.18	1.06	0.39	↓^∗^/1.19
Succinate	2.37 (s)	2.57	0.63	↓^∗∗^/1.12	1.84	0.57	↓^∗∗^/1.23	—	—	—/1.13
Citrate	2.54 (d), 2.66 (d)	—	—	—/1.01	2.92	0.61	↓^∗∗^/1.21	2.79	0.54	↓^∗∗^/1.17
Aspartate	2.87 (m), 2.94 (m)	1.49	0.74	↓^∗∗^/1.40	1.51	0.76	↓^∗∗^/1.62	—	—	—/1.09
Choline	3.20 (s)	2.31	0.54	↑^∗∗^/1.23	2.1	0.48	↑^∗∗^/1.20	—	—	—/1.02
Proline	3.36 (m)	—	—	—/1.03	4.03	0.65	↓^∗∗^/1.20	3.41	0.44	↓^∗∗^/1.26
Glycine	3.57 (s)	2.62	0.48	↑^∗∗^/1.19	2.21	0.52	↑^∗∗^/1.46	—	—	—/1.04
Glucose	3.24 (q), 3.48 (t), 3.90 (q), 3.54 (t), 3.71 (t), 3.83 (t)	2.49	0.52	↓^∗∗^/1.25	1.87	0.67	↑^∗∗^/1.53	3.46	0.47	↑^∗∗^/1.31
Serine	3.84 (m), 3.96 (m)	1.57	0.68	↑^∗∗^/1.32	1.19	0.59	↑^∗∗^/1.18	1.68	0.60	↑^∗∗^/1.23
Tyrosine	6.90 (d), 7.20 (d)	—	—	—/1.01	1.01	0.52	↓^∗∗^/1.19	1.12	0.54	↓^∗∗^/1.22
NAc	2.03 (s)	1.09	0.62	↓^∗∗^/1.27	—	—	—/1.01	2.93	0.74	↑^∗∗^/1.21
PUFA	2.76-2.83 (bra)	1.1	0.41	↑^∗^/1.18	—	—	—/1.04	1.46	0.43	↓^∗∗^/1.37
Glycerol	3.61 (m), 3.65 (m)	1.74	0.61	↓^∗∗^/1.32	—	—	—/1.07	—	—	—/1.05
Lysine	1.45 (m), 1.71 (m), 1.89 (m), 3.02 (t), 3.75 (t)	—	—	—/1.02	—	—	—/1.03	1.04	0.49	↑^∗∗^/1.26

s: single; d: doublet; dd: doublet of doublet; t: triplet; q: quartet; m: multiplet; bra: broad peak; ↑: increase; ↓: decrease; —: no significant change. ^∗^ *p* < 0.05; ^∗∗^ *p* < 0.01.

## Data Availability

The data used to support the findings of this study are available from the corresponding authors upon request.

## Supplementary Materials

Supplementary Materials Supporting information supporting tables: Table S1: main demographic and clinical features of the participants. Table S2: the major parameters of CV-AVOVA in the OPLS-DA model of colorectal polyp vs. control. Table S3: the major parameters of CV-AVOVA in the OPLS-DA model of CRC vs. control. Table S4: the major parameters of CV-AVOVA in the OPLS-DA model of CRC vs. colorectal polyp. Table S5: the major parameters in the ROC curve of acetate/glycerol. Table S6: the major parameters in the ROC curve of lactate/citrate. Table S7: main demographic and clinical features of the participants from the validation samples. Supporting figures: Figure S1: the typical 1H-NMR spectra of serum samples from colorectal cancer patients (red), colorectal polyp patients (blue), and healthy controls (black). Figure S2: the typical TOCSY spectrum of the control serum sample. Figure S3: PLS-DA score plots derived from NMR spectra of the serum samples and the corresponding validation plots of PLS-DA models, generated from the permutation tests that were randomly permuted 600 times. Figure S4: the corresponding validation plots of OPLS-DA models, generated from the permutation tests that were randomly permuted 600 times. Figure S5: the results of SVM classifier in the validation set.
