# Supplementary material for: Metabolomics Analysis in Serum from Patients with Colorectal Polyp and Colorectal Cancer by 1H-NMR Spectrometry
Source: Dis Markers. 2019 Apr 7;2019:3491852. doi: 10.1155/2019/3491852 (PMC6476004; doi:10.1155/2019/3491852)
Supplement: Supplementary Materials — Supporting information supporting tables: Table S1: main demographic and clinical features of the participants. Table S2: the major parameters of CV-AVOVA in the OPLS-DA model of colorectal polyp vs. control. Table S3: the major parameters of CV-AVOVA in the OPLS-DA model of CRC vs. control. Table S4: the major parameters of CV-AVOVA in the OPLS-DA model of CRC vs. colorectal polyp. Table S5: the major parameters in the ROC curve of acetate/glycerol. Table S6: the major parameters in the ROC curve of lactate/citrate. Table S7: main demographic and clinical features of the participants from the validation samples. Supporting figures: Figure S1: the typical 1H-NMR spectra of serum samples from colorectal cancer patients (red), colorectal polyp patients (blue), and healthy controls (black). Figure S2: the typical TOCSY spectrum of the control serum sample. Figure S3: PLS-DA score plots derived from NMR spectra of the serum samples and the corresponding validation plots of PLS-DA models, generated from the permutation tests that were randomly permuted 600 times. Figure S4: the corresponding validation plots of OPLS-DA models, generated from the permutation tests that were randomly permuted 600 times. Figure S5: the results of SVM classifier in the validation set. [file 3491852.f1.pdf]

Supplementary Material

Metabolomics analysis in serum from patients with colorectal polyp and colorectal cancer by ^{1}H-NMR spectrometry

Jinping Gu, Yaqing Xiao, Dan Shu, Xianrui Liang, Xiaomin Hu, Yuanyuan Xie,

Donghai Lin, Hua Li

5 Supplementary Figures

7 Supplementary Tables

Table S1. Main demographic and clinical features of the participants.

	healthy controls	Colorectal polyp patients	CRC patients
Number	38	32	40
Age (Median, Range)	55, 35-77	57, 26-86	60, 25-82
Sex (Male / Female)	21/17	20/12	27/13
Tumor stage			
Stage I	/	/	7
Stage II	/	/	9
Stage III	/	/	14
Stage IV	/	/	10

Table S2. The major parameters of CV-AVOVA in the OPLS-DA model of Colorectal polyp vs. Control

	SS	DF	MS	F	p	SD
Total corr.	69	69	1			1
Regression	54.262	4	13.5655	59.8287	4.32E-21	3.68314
Residual	14.738	65	0.226739			0.476171

Table S3. The major parameters of CV-AVOVA in the OPLS-DA model of CRC vs. Control

	SS	DF	MS	F	p	SD
Total corr.	77	77	1			1
Regression	66.116	4	16.529	110.861	3.13E-30	4.06559
Residual	10.884	73	0.149096			0.38613

Table S4. The major parameters of CV-AVOVA in the OPLS-DA model of CRC vs. Colorectal polyp

	SS	DF	MS	F	p	SD
Total corr.	71	71	1			1
Regression	26.3314	4	9.58284	19.87383	2.43E-06	2.56571
Residual	24.6686	67	0.366696			0.416514

Table S5. The major parameters in the ROC curve of acetate/glycerol

	Threshold	Sensitivity	Specificity	AUC	Accuracy	PPV	NPV
Colorectal polyp vs. Control	0.3145	0.8125	0.7895	0.8310	80.00%	76.47%	83.33%
Colorectal polyp vs. CRC	0.3417	0.5750	0.8158	0.7130	69.23%	76.67%	64.58%
CRC vs. Control	0.2997	0.7188	0.6250	0.6690	66.67%	60.53%	73.53%

Table S6. The major parameters in the ROC curve of lactate/citrate

	Threshold	Sensitivity	Specificity	AUC	Accuracy	PPV	NPV
Colorectal polyp vs. Control	4.7432	0.7813	0.7368	0.8210	75.71%	71.43%	80.00%
Colorectal polyp vs. CRC	6.0213	0.7188	0.6750	0.7720	69.44%	63.89%	75.00%
CRC vs. Control	4.7778	0.7750	0.7368	0.8270	75.64%	75.61%	75.68%

Table S7. Main demographic and clinical features of the participants from the validation samples

	healthy controls	Colorectal polyp patients	CRC patients
Number	8	8	8
Age (Median, Range)	40, 35-47	47, 44-66	50, 48-68
Sex (Male / Female)	4/4	5/3	6/2
Tumor stage			
Stage I	/	/	2
Stage II	/	/	5
Stage III	/	/	1
Stage IV	/	/	/

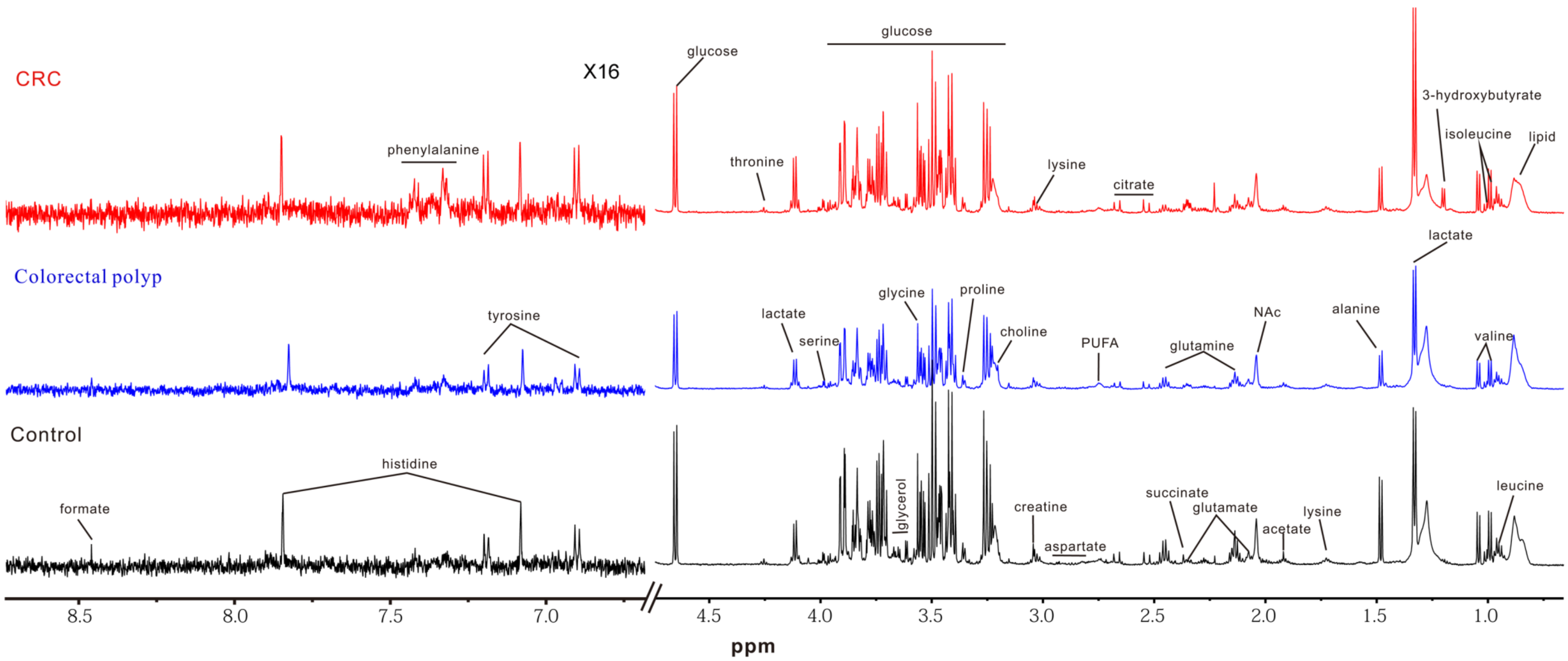

Fig. S1. The typical ^{1}H NMR spectra of serum samples from colorectal cancer patients (red), colorectal polyp patients (blue) and healthy controls (black) , respectively.

*NAc, N-acetyl glycoproteins; PUFA, polyunsaturated fatty acid.

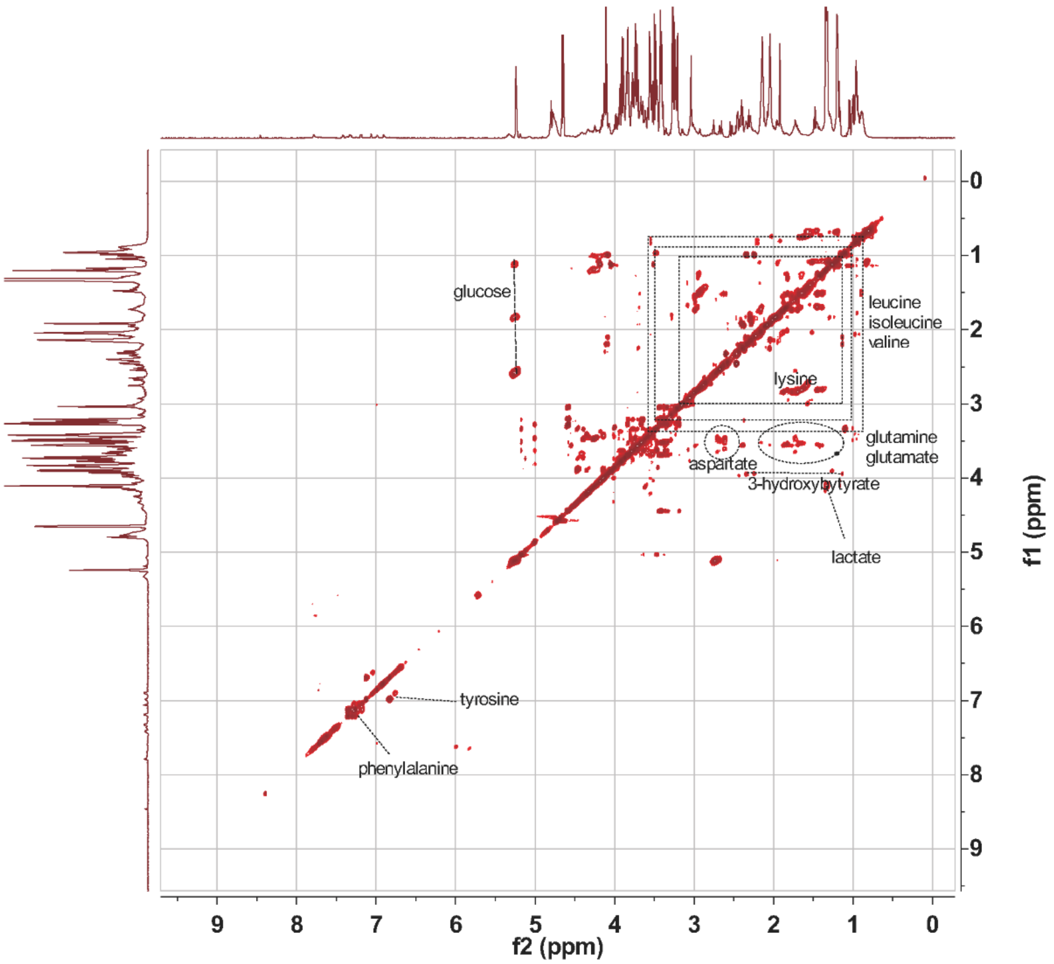

Fig. S2. The typical TOCSY spectrum of control serum sample

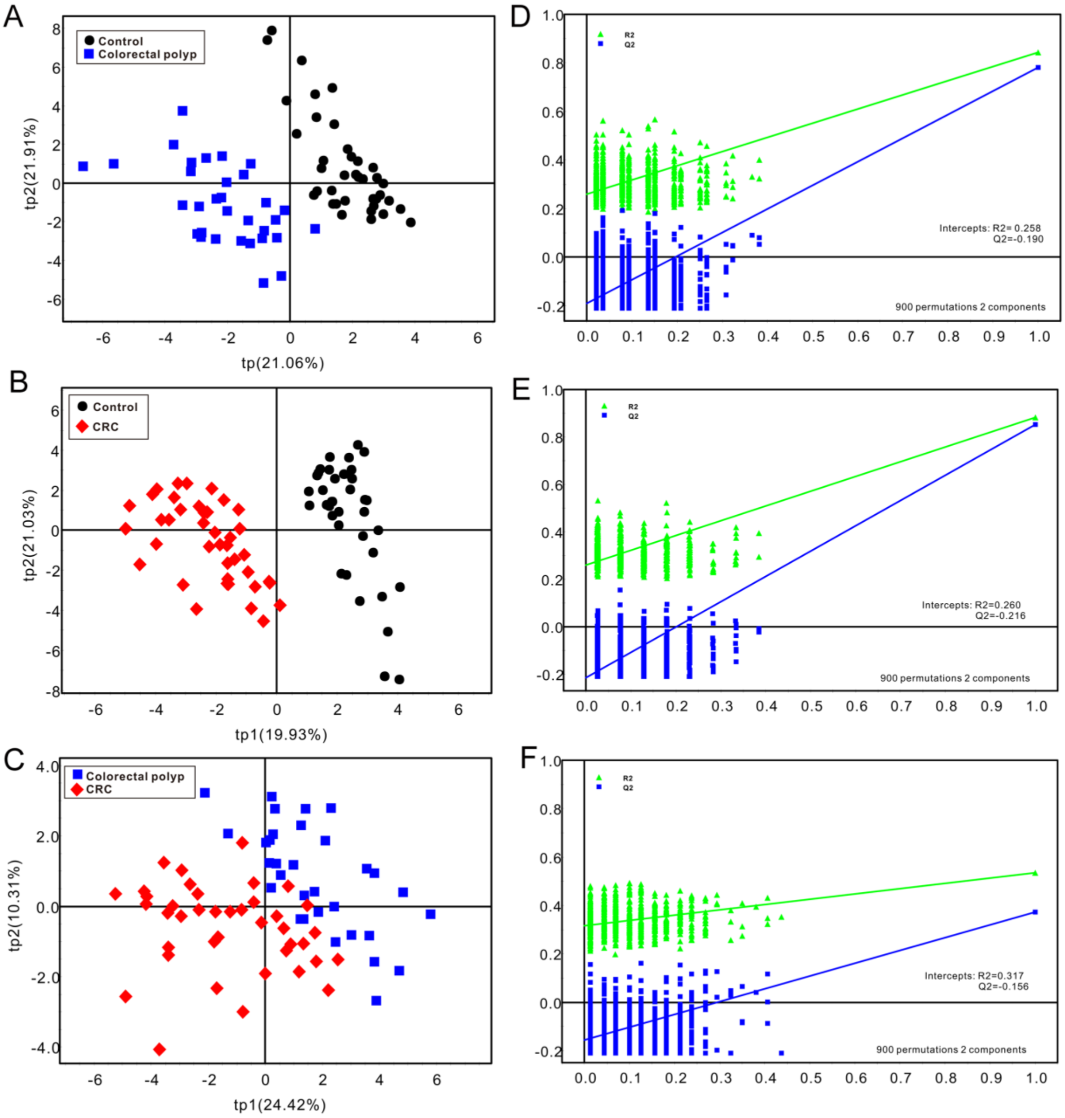

Fig. S3. PLS-DA scores plots derived from NMR spectra of the serum samples (A, B, C) and the corresponding validation plots of PLS-DA models, which generated from the permutation tests that were randomly permuted 600 times (D, E, F). (A, D) Colorectal polyps vs. Controls; (B, E) CRC vs. Control; (C, F) CRC vs. Colorectal polyps.

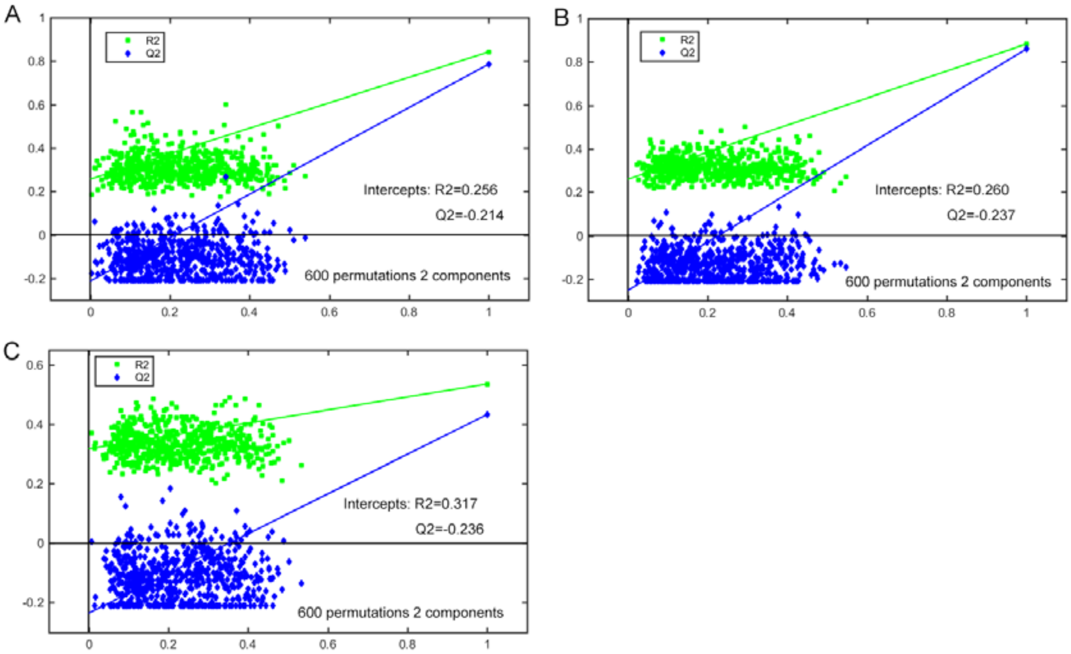

Fig. S4. The corresponding validation plots of OPLS-DA models, which generated from the permutation tests that were randomly permuted 600 times (A) Colorectal polyps vs. Controls; (B) CRC vs. Control; (C) CRC vs. Colorectal polyps.

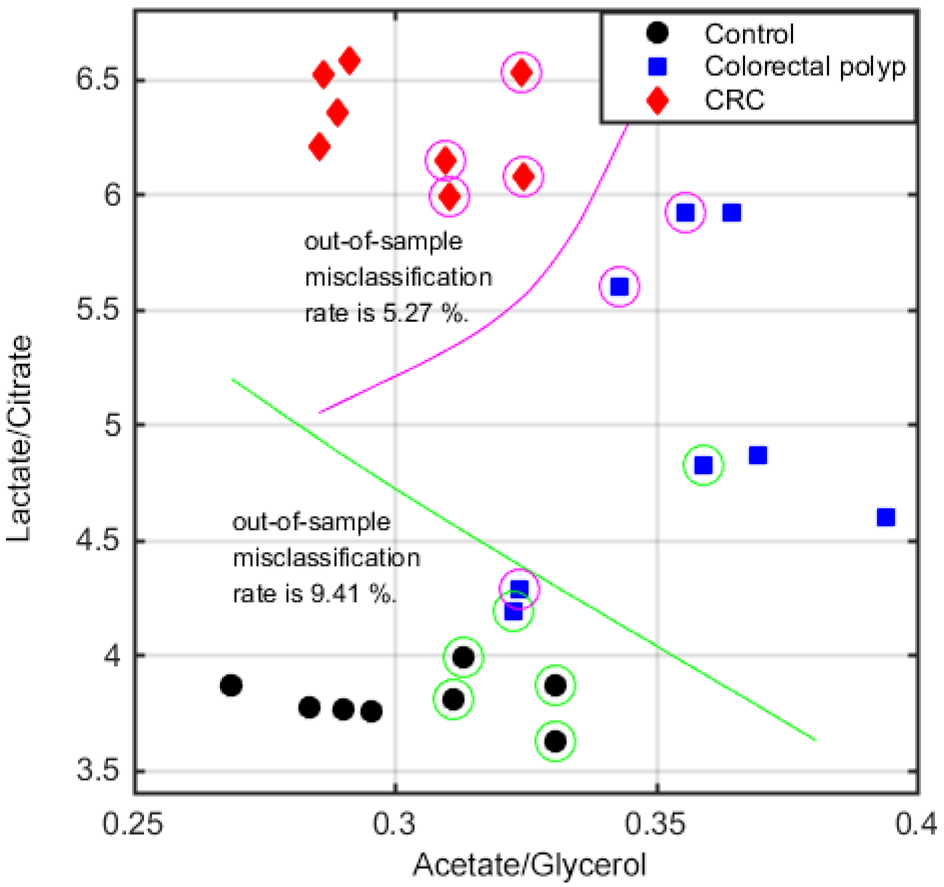

Fig. S5. The results of SVM classifier in the validation set
